# Multiplexed Complementary Signal Transmission for a Self‐Regulating Artificial Nervous System

**DOI:** 10.1002/advs.202205155

**Published:** 2022-11-27

**Authors:** Young Jin Choi, Dong Gue Roe, Yoon Young Choi, Seongchan Kim, Sae Byeok Jo, Hwa Sung Lee, Do Hwan Kim, Jeong Ho Cho

**Affiliations:** ^1^ Department of Chemical and Biomolecular Engineering Yonsei University Seoul 03722 Republic of Korea; ^2^ School of Electrical and Electronic Engineering Yonsei University Seoul 03722 Republic of Korea; ^3^ Department of Mechanical Science and Engineering University of Illinois at Urbana−Champaign Urbana IL 61801 USA; ^4^ SKKU Advanced Institute of Nanotechnology (SAINT) Sungkyunkwan University Suwon 16419 Republic of Korea; ^5^ School of Chemical Engineering SKKU Institute of Energy Science and Technology (SIEST) Sungkyunkwan University (SKKU) Suwon 16419 Republic of Korea; ^6^ Department of Materials Science and Chemical Engineering Hanyang University Ansan 15588 Republic of Korea; ^7^ Department of Chemical Engineering Hanyang University Seoul 04763 Republic of Korea

**Keywords:** artificial nervous system, healthcare, multi‐level regulation, Schottky barrier transistor, signal multiplexing

## Abstract

Neuromorphic engineering has emerged as a promising research field that can enable efficient and sophisticated signal transmission by mimicking the biological nervous system. This paper presents an artificial nervous system capable of facile self‐regulation via multiplexed complementary signals. Based on the tunable nature of the Schottky barrier of a complementary signal integration circuit, a pair of complementary signals is successfully integrated to realize efficient signal transmission. As a proof of concept, a feedback‐based blood glucose level control system is constructed by incorporating a glucose/insulin sensor, a complementary signal integration circuit, an artificial synapse, and an artificial neuron circuit. Certain amounts of glucose and insulin in the initial state are detected by each sensor and reflected as positive and negative amplitudes of the multiplexed presynaptic pulses, respectively. Subsequently, the pulses are converted to postsynaptic current, which triggered the injection of glucose or insulin in a way that confined the glucose level to a desirable range. The proposed artificial nervous system demonstrates the notable potential of practical advances in complementary control engineering.

## Graphical abstract

This paper presents a complementary control‐based homeostatic regulating artificial nervous system by adopting a barrier‐tunable SBT. A blood glucose level regulation system is constructed by incorporating glucose/insulin sensors, SIC, AS, and ANC, which is capable of multi‐level regulation. The proposed artificial nervous system demonstrates the notable potential of practical advances in complementary control engineering.

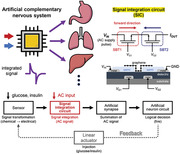

## 1 Introduction

The regulatory system in the human body is governed by the autonomic nervous system, which controls a broad range of unconscious response of internal organs and muscles such as cardiac function, respiration, vasomotor, and pupil reflex. The autonomic nervous system consists of three divisions: sympathetic nervous system, parasympathetic nervous system, and enteric nervous system. Among these systems, sympathetic nervous system and parasympathetic nervous system play a role of maintaining homeostasis through opposing actions, that is, the antagonism.^[^ ^1^, ^2^, ^3^ ^]^ One of the most representative examples of the antagonism is the blood glucose level regulation; two antagonistic hormones, insulin, and glucagon are secreted autonomously to reduce and raise the blood glucose levels, respectively, upon the stimuli induced by the changes in the blood glucose level. However, if a body can no longer produce insulin or becomes resistant to insulin, a serious metabolic disorder from unregulated blood glucose level may occur, which is termed diabetes. Diabetes is the direct cause of hyperglycemia, which is followed by multitudes of life‐threatening complications such as cardiovascular disease and diabetic neuropathy.^[^ ^4^, ^5^, ^6^ ^]^ For managing the diabetes and diabetic symptoms, an external insulin infusion can be administered in the form of an injection or an oral medicine. However, the amount and frequency of the infusion must be determined with a high precision in order to avoid serious adverse effects such as hypoglycemia. Therefore, a development of real‐time multi‐level insulin control system is imperative to achieve a high precision healthcare.

In such aspect, a feasible breakthrough can be found in the field of artificial nervous system. The artificial nervous system essentially mimics the efficient signal processing of biological neurons, which thus has been widely studied as a method to overcome the various chronic limitations of conventional CMOS based systems.^[^ ^7^, ^8^, ^9^, ^10^, ^11^, ^12^, ^13^, ^14^, ^15^, ^16^, ^17^, ^18^, ^19^, ^20^ ^]^ Especially, the neuromorphic system has a strong advantage in achieving portable, human‐friendly, and ubiquitous healthcare environments, owing to the beneficial traits in power consumption and device integration of synaptic processing. Beyond the simple mimicry of synaptic operations, recently, the primary focus of neuromorphic systems research has shifted to a more systematic simulation of the whole biological systems such as stimulus‐response mechanisms.^[^ ^21^, ^22^, ^23^, ^24^, ^25^, ^26^, ^27^, ^28^, ^29^, ^30^, ^31^, ^32^, ^33^, ^34^, ^35^, ^36^, ^37^, ^38^ ^]^ For example, Lee's group designed an artificial afferent nervous system that can be directly connected to the motor neurons of the leg of a discoid cockroach.^[^ ^35^ ^]^ The authors demonstrated that the artificial nervous system can successfully control the leg actuation through the application of analog external stimuli. The analog‐based non‐Boolean processing of artificial nervous system is capable of highly complex functionalities such as multi‐level response and homeostatic regulation. In particular, multi‐level response system that divides a single action into several actions of a weaker level could easily be implemented by placing several levels of action thresholds in analog signals by using the artificial nervous system. The artificial nervous system can further enable an efficient use of complex inputs such as complementary pulse signals of both positive and negative amplitudes, for which several signal channels and corresponding processors should be arranged parallelly in the existing Boolean logic systems. Furthermore, the capability of complex signal processing in artificial nervous systems can even embrace the information in the multiplexed form of amplitude, width, and frequency of the input signal pulses, through which they manifest themselves as a feasible candidate to achieve breakthroughs in the complexity and efficiency of signal processing in healthcare technology.

In this manuscript, we present an artificial complementary nervous system capable of facile and sophisticated homeostatic regulations of blood glucose levels. A multi‐level response method associated with the complementary pulse inputs is implemented by using a Schmitt‐trigger‐based multi threshold artificial neuron circuit (ANC) and a Schottky‐barrier–transistor (SBT)‐based signal integration circuit (SIC). The systems architecture is completed by incorporating insulin/glucose sensors, an artificial synapse (AS), SIC, and ANC. In this system, the glucose and insulin levels are first converted into electrical signals by sensors connected to the SIC. The signals are then reflected as positive and negative amplitudes of the complementary pulse voltages through two SBTs connected in series. The amplitudes of complementary signals were modulated through tunable diode characteristics of SBTs. The resulting multiplexed voltage pulses are transmitted to the AS to generate postsynaptic current (PSC), which becomes an analog indicator of the glucose level. Finally, the multi‐level blood glucose/insulin regulation system is realized by controlling the injection of insulin according to the level of the PSC through the ANC. Moreover, since SBT and AS could take roles of the signal processing and computation units in the conventional regulation system by a single device, the total complexity of the system was decreased. With the successful demonstration of multi‐level regulation capabilities, the proposed artificial complementary nervous system can broaden the horizon of artificial nervous system by incorporating a new possibility of the sophisticated multiplexed signal processing.

## 2 Results and Discussion

**Figure 1:**
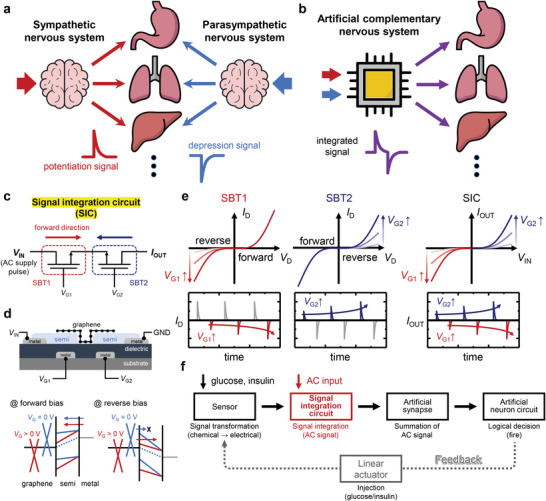
a) Schematic of complementary sympathetic nervous system/parasympathetic nervous system‐based biological antagonistic regulation system. b) Schematic of artificial complementary nervous system that uses integrated complementary signals. c) Schematic circuit diagram of signal integration circuit (SIC) based on Schottky‐barrier–transistors (SBTs) connected in series in opposite directions. d) Schematic cross‐sectional structure of SIC and its band diagram at the forward and reverse bias conditions. e) *V*_G_‐dependent output characteristics of an individual SBT and SIC. f) Operational diagram of glucose level‐regulation system via artificial complementary nervous system.

**Figure** **1**a depicts a biological control system based on the complementary signals of sympathetic nervous system/parasympathetic nervous system. The combination of these systems results in complementary stimuli delivered to organs, such as promotion‐inhibition and dilation‐contraction. Although a biological control system can effectively control the internal environment of the human body through complementary signals, the presence of sympathetic nervous system and parasympathetic nervous system necessitates two independent signal transmission channels, which leads to a high system complexity. In contrast, the proposed artificial complementary nervous system combines the two complementary signals into an integrated signal before it reaches the associated organs, thereby simplifying the transmission system to realize efficient signal transmission, as shown in Figure 1b.

The operating characteristics of the SIC are of significance in integrating the two complementary signals: Changes in individual signal types can be discerned to reflect each signal in the form of positive and negative amplitudes of the pulses. Figure 1c shows a circuit diagram of the SIC that integrates two complementary signals into voltage pulses. The circuit contains two SBTs connected in series in opposite directions. When the complementary input signals (*V*_G1_ and *V*_G2_) to be integrated enter the gate of each SBT, an AC supply pulse applied to the drain (*V*_IN_) is converted to an asymmetric AC output pulse, the positive and negative amplitudes of which are adjusted according to each input signal. The varying amplitude of the two signs of the asymmetric AC can be attributed to the tunable diode behavior of the SBTs. The upper panel of Figure 1d and Figure S1, Supporting Information show schematic structure and optical microscopy image of two SBTs connected in series, respectively. Each SBT has a graphene/n‐type semiconductor/metal heterojunction (Figure S2, Supporting Information). Such structure allows modulation of the Schottky barrier, as shown in the lower panel of Figure 1d. In the forward bias mode, the injection of electrons from the metal into the semiconductor is not affected because the work function change of the metal owing to *V*_G_ is negligible. In contrast, in the reverse mode, the electron injection can be adjusted based on *V*_G_ because the Schottky barrier at the graphene/semiconductor interface can be either raised or lowered by applying negative or positive *V*_G_, respectively.^[^ ^39^, ^40^, ^41^, ^42^ ^]^ The voltage polarity of the SBTs determines the sign of the output current that can be adjusted, as shown in the schematic output characteristics of SBTs and SIC (Figure 1e). In the case of SBT1 (left panel of Figure 1e), the reverse mode occurs in the negative drain voltage (*V*_D_) region. Thus, the positive component of the AC supply pulse yields an output with a constant and high‐level positive amplitude, whereas the negative supply component results in *V*_G_‐dependent negative output pulses. In the case of SBT2 (middle panel of Figure 1e), the bias condition is reversed: The positive component of the AC supply pulse solely contributes to the positive amplitude of the asymmetric AC pulse, which varies according to *V*_G_. When these two SBTs are connected in series (right panel of Figure 1e), the SBT with the higher resistance determines the amplitude of the output pulse for a given AC supply pulse. As a result, the negative and positive amplitudes of the resulting asymmetric AC output pulse can be modulated only through *V*_G1_ and *V*_G2_, respectively.

Figure 1f shows the operational diagram of regulation of blood glucose level by the artificial complementary nervous system. First, the glucose and insulin sensors detect each target substance to convert it into an electrical signal. Subsequently, each signal is integrated to voltage pulses including the information of both the glucose and insulin by SIC. Next, the integrated signal enters the AS, which potentiates and depresses the PSC according to the positive and negative amplitudes of the input signal, respectively. When the level of PSC exceeds the set standard, a logical decision is made to suppress the change in the PSC by the ANC. Consequently, a negative feedback is achieved by transmitting the signal to the actuators that inject glucose or insulin to maintain the glucose level in a desirable range.

**Figure 2:**
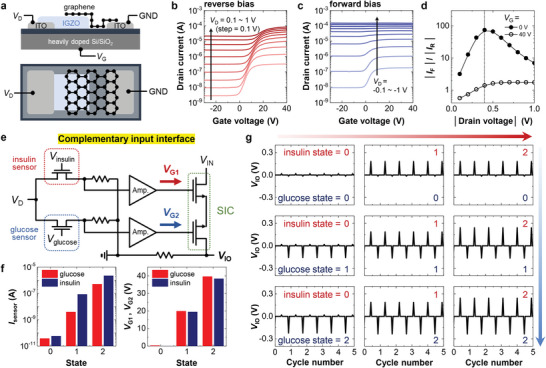
a) Schematic structure of a graphene/IGZO SBT. b,c) Transfer characteristics of an SBT under b) reverse and c) forward bias conditions. d) Rectification ratio (|*I*_F_|/|*I*_R_|) of an SBT as a function of *V*_D_ at *V*_G_ = 0 and 40 V. e) Schematic circuit diagram of a complementary input interface composed of a glucose sensor, an insulin sensor, and an SIC. f) *I*_sensor_, *V*_G1_, and *V*_G2_ as a function of the glucose and insulin states. g) *V*_IO_ under various glucose and insulin states.

**Figure** **2**a shows the cross‐section and top‐view schematic of a single SBT. First, an indium‐tin‐oxide (ITO) drain electrode and indium‐gallium‐zinc‐oxide (IGZO) channel were sputtered onto a heavily‐doped Si/SiO_2_ substrate. Subsequently, the monolayer graphene was transferred and patterned onto the IGZO channel layer to form the Schottky barrier between the layers (Figures S3 and S4, Supporting Information). Figure 2b,c show transfer curves of the SBT under reverse *V*_D_ ranging from +0.1 to +1.0 V in 0.1 V increments and forward *V*_D_ ranging from −0.1 to −1.0 V in −0.1 V increments, respectively (also see the band diagram and output curve are presented in Figures S5 and S6, Supporting Information, respectively). In both reverse and forward bias modes, large *V*_D_‐dependent current modulations due to the change of charge transport mechanism were observed^[^ ^42^ ^]^, but the *V*_G_‐dependencies showed asymmetric current modulation of typical SBTs, with high uniformities (Figure S7, Supporting Information). In the reverse bias mode, the maximum on–off current ratio of 156 was observed when *V*_D_ was +0.2 V. However, in the forward bias mode, the on–off current ratio did not exceed 20 under the same *V*_D_ range (Figure S8, Supporting Information). The rectification ratio, which is defined as the ratio of the forward current to the reverse current at *V*_G_ = 0 and 40 V as a function of *V*_D_ is plotted in Figure 2d and Figure S9, Supporting Information. When *V*_G_ = 0 V, the highest rectification ratio was observed at *V*_D_ = 0.4 V, while the ratio approximately converged to 1 under *V*_G_ = 40 V. For an SBT that exhibits a high rectification ratio, the contribution of the forward mode SBT to the overall output current of the system in which two SBTs in forward and reverse modes are connected in series is negligible. Because the forward mode current of the adopted SBT does not overwhelm the reverse mode current, it is necessary to design the system to ensure that the difference between the forward and reverse mode currents is maximized. Therefore, *V*_D_ = 0.4 V, which corresponds to the maximum rectification ratio, was selected as the amplitude of the AC supply pulse of the SIC.

Figure 2e shows the circuit diagram of the complementary input interface, which consists of a glucose sensor, an insulin sensor, and a SIC that incorporates the electric signals from the two sensors. Transistor‐type sensors are fabricated for detecting the glucose/insulin level. IGZO was used as a benchmark sensing channel despite its lack of selectivity because the current level can be varied by several orders according to the amounts of glucose or insulin solutions (Figures S10 and S11, Supporting Information). The current levels of the sensors were varied by the area of the droplet that contacted to the IGZO channel. The high on–off current ratio of transistor‐type sensors allows three glucose/insulin levels to be distinguished. The amounts of glucose and insulin detected by each sensor are first converted to the corresponding *I*_sensor_, as shown in the left panel of Figure 2f. States “0,” “1,” and “2” denote the number of glucose or insulin doses dropped on each sensor, respectively. Next, *I*_sensor_ was converted to a voltage signal via a voltage divider followed by voltage amplification. By selecting the proper reference resistance and voltage gain of the amplifier, it is possible to adjust *I*_sensor_ to the intended voltage level. In the considered case, states “0”, “1”, and “2” correspond to approximately 0, 20, and 40 V, respectively, as shown in the right panel of Figure 2f. Finally, the amplified voltage was applied to the gate of each SBT connected in series in opposite directions while the AC supply voltage, with an amplitude of 0.4 V and frequency of 2 Hz, was applied to the drain of the SBT. During the positive amplitude phase, the SBTs connected to the glucose and insulin sensors were in the reverse and forward modes, respectively. Therefore, the amplitude of the positive composition of the integrated output voltage (*V*_IO_) pulse was determined by *V*_G1_ of the SBT connected to the glucose sensor (i.e., the state of glucose). Likewise, the negative composition of the *V*_IO_ pulse was regulated by the state of insulin. As a proof of concept, the *V*_IO_ pulse according to the states of glucose and insulin were measured, as shown in Figure 2g. As the glucose and insulin state increased, the amplitude of the positive and negative components of the *V*_IO_ pulse increased, respectively, and the state of each substance independently contributed to the amplitude of the *V*_IO_ pulse.

**Figure 3:**
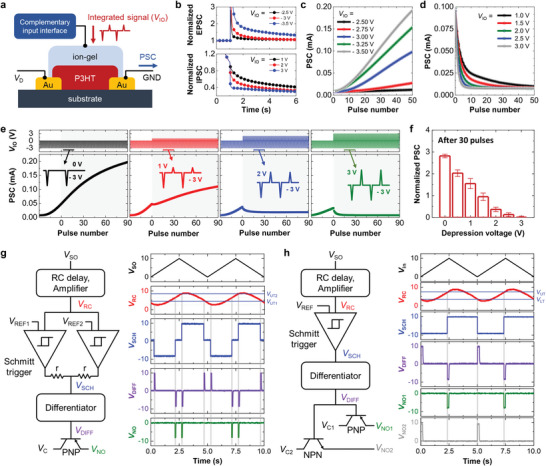
a) Schematic structure of P3HT/ion–gel AS. b) Normalized EPSC and IPSC of AS under various *V*_IO_ pulses. c) Long‐term plasticity behavior of AS with *V*_IO_ = −2.5 to −3.5 V. d) Long‐term depression behavior with *V*_IO_ = +1.0 to +3.0 V. e) PSC of AS according to the positive amplitude of the integrated pulses (*V*_0,3_, *V*_1,3_, *V*_2,3_, and *V*_3,3_) after prepotentiation with 30 negative pulses (*V*_IO_ = −3 V, *W* = 40 ms). f) Normalized PSC as a function of the positive amplitude of pulses after 30 integrated pulses. g) Schematic circuit diagram of double‐negative spike generation ANC and resulting output signals of double‐negative spike generation ANC at each electrical component with triangular *V*_SO_ (*V*_Amp_ = 10 mV, *f* = 0.2 Hz). h) Schematic circuit diagram of the complementary spike generation ANC. and resulting output signals of the complementary spike generation ANC at each electrical component with triangular *V*_in_ (*V*_Amp_ = 10 mV, *f* = 0.2 Hz).

In order to regulate the levels of glucose and insulin within a desirable range, it is necessary to first understand the synaptic behavior of the AS induced by the *V*_IO_ pulse input. **Figure** **3**a depicts the structural schematic of the AS and *V*_IO_ entering the AS. For the fabrication of the AS, a poly(3‐hexylthiophene) (P3HT) and an ion–gel were patterned onto the substrate with Au electrode patterns (Figure S12, Supporting Information). Detailed working mechanism of the P3HT/ion–gel AS is as follows: Negative *V*_IO_ pulses drive the penetration of [PF_6_]^−^ anions in the ion–gel into the free volume of the P3HT channel layer and facilitate the doping of the P3HT channel with the anions to increase the PSC. Although the penetrated anions gradually diffuse out from the channel in the absence of the negative *V*_IO_ pulses, the residual anions in the P3HT prevent a drastic reduction in the PSC, resulting in retentive current characteristics. Furthermore, the penetrated [PF_6_]^−^ ions are extracted by positive *V*_IO_ pulses, and current retention occurs during the absence of *V*_IO_. Such retentive characteristics of the P3HT/ion–gel layer results in the synaptic behavior.^[^ ^24^ ^]^ Figure 3b shows the normalized EPSC and IPSC of the P3HT/ion–gel AS. *V*_IO_ pulses with magnitude from +1 to +3 V and a width of 40 ms were applied to the ion–gel, and clear EPSC and IPSC responses are observed at a *V*_D_ of 0.5 V. Moreover, the AS exhibited reliable long‐term potentiation (Figure 3c) as well as long‐term depression behaviors (Figure 3d). Long‐term plasticity due to the retentive ion movement in the P3HT channel was observed up to 50 consecutive pulses, and the changes in the PSC became larger as the pulse magnitude increased. In addition, the thermal stability of the AS was evaluated in Figure S13, Supporting Information.

Based on the fundamental synaptic performance discussed in the previous sections, the PSC change of the P3HT/ion–gel AS by *V*_IO_ pulses was clarified, as shown in Figure 3e. Prior to applying *V*_IO_ pulses having both positive and negative peak components, the current potentiation by negative pulses with a magnitude of −3 V (*V*_0,3_) was investigated. As shown in the first panel (black line), gradual PSC increase was observed. Next, the synaptic device subjected to 30 *V*_0,3_ pulses in advance was exposed to continuous *V*_IO_ pulses, with the magnitude of the positive peak component set as +1 (red line), +2 (blue line), and +3 V (green line), and the magnitude of the negative peak component set as −3 V (denoted as *V*_1,3_, *V*_2,3_, and *V*_3,3_, respectively). Note that, the pulse numbers in Figure 3e were counted after applications of the 30 *V*_0,3_ pulses were completed, unlike Figure 3c. Compared to *V*_0,3_, *V*_1,3_ pulses mitigated the increase in PSC, as indicated by the gray shaded region. In the case of *V*_2,3_, the PSC decreased from the initial state but maintained a certain current level even when the number of pulses increased. The application of *V*_3,3_ further decreased the PSC level, reaching nearly zero after nine pulses. Additional experimental results regarding the PSC changes under positive peak components of 0.5, 1.5, and 2.5 V along with 0, 1, 2, and 3 V are shown in Figure S14, Supporting Information. Figure S15, Supporting Information shows the derivative of PSC with respect to the number of pulses. When the number of pulses is sufficiently large and the positive peak component is less than 1 V, the derivative was positive; above 2 V, the value was close to zero. Figure 3f indicates the level of normalized PSC according to the magnitude of the positive peak component. As the depression voltage increased from 0 to 3 V, the PSC after 30 pulses decreased from 2.81 to 9.4 × 10^−3^A. These results indicate that the PSC behavior can be controlled by modulating the positive and negative amplitudes of the *V*_IO_ pulses.

To construct a glucose level‐regulating system, a Schmitt‐trigger‐based ANC that provides feedback regarding the glucose and insulin levels was designed. The ANC consists of four electrical parts: i) A resistor–capacitor delay and a noninverting amplifier that reduces the intensity of the spike component of the synaptic output voltage, (*V*_SO_, ANC‐entering voltage converted from PSC by a voltage divider, see Figure S16, Supporting Information) and amplifies the mitigated *V*_SO_ (i.e., *V*_RC_), respectively; ii) a Schmitt trigger that outputs the voltages (*V*_SCH_) for the *V*_RC_ according to two set thresholds of *V*_UT_ and *V*_LT_; iii) a differentiator that converts the square wave of *V*_SCH_ into a pulse wave (*V*_DIFF_); and iv) a PNP and NPN bipolar‐junction transistor (BJT) that selectively transfers negative or positive pulses (ANC output voltage, *V*_NO_). These *V*_NO_ signals induce an injection of glucose and insulin to adjust the glucose level. Since the number and signs of the output pulses depend on the number of Schmitt triggers and the type of BJT, a variety of glucose‐level‐regulating systems can be implemented by varying these two electrical parameters. Figure 3g shows the simplified circuit diagram and output voltages obtained after traversing each electrical component of a double‐negative spike generation ANC having two Schmitt triggers with different reference voltages (*V*_REF_) and a PNP BJT. Utilization of two Schmitt triggers connected in parallel allows three distinct levels of *V*_SCH_ to be implemented, thereby yielding double *V*_DIFF_ spikes. Moreover, the PNP BJT selectively passes only negative pulses. As the input, triangular pulses (*V*_Amp_ = 10 mV, *f* = 0.2 Hz) were applied with a supply voltage of ± 10 V and *V*_REF_ of 4.5 and 6.5 V. The two pairs of thresholds set according to the Schmitt triggers were *V*_UT_ = 6.5 V, *V*_LT_ = 2.5 V and *V*_UT_ = 8.5 V, *V*_LT_ = 4.5 V for the double *V*_DIFF_ spike. *V*_SCH_ exhibits an upshift and downshift when *V*_RC_ coincides with *V*_UT_ and *V*_LT_, yielding a negative and positive pulse, respectively; this phenomenon is followed by the filtering of the positive pulses by the PNP BJT. In this manner, double‐negative *V*_NO_ spikes capable of inducing a steeper increase in the glucose level could be generated. In addition to the double‐negative spike generation ANC, a complementary spike generation mode glucose‐level‐regulating system was designed by replacing the pair of Schmitt‐triggers with a single one and adding a NPN BJT. The operation principle of this system is identical to that of the abovementioned system, except for the *V*_DIFF_ spike generation and pulse filtering being performed by the BJT, as shown in the simplified circuit diagram of the complementary spike generation ANC (Figure 3h). By using one Schmitt‐trigger (*V*_UT_ = 7.5 V, *V*_LT_ = 3.5 V) and both types of BJT, a single *V*_DIFF_ spike in both positive and negative can be obtained. The detailed circuit diagrams of the ANCs and information on the Schmitt‐trigger and the differentiator are shown in the supporting information (Figures S17–S19, Supporting Information). The advantage of this system is that both glucose and insulin injection can be facilitated in a manner that prevents a radical change in the glucose level.

**Figure 4:**
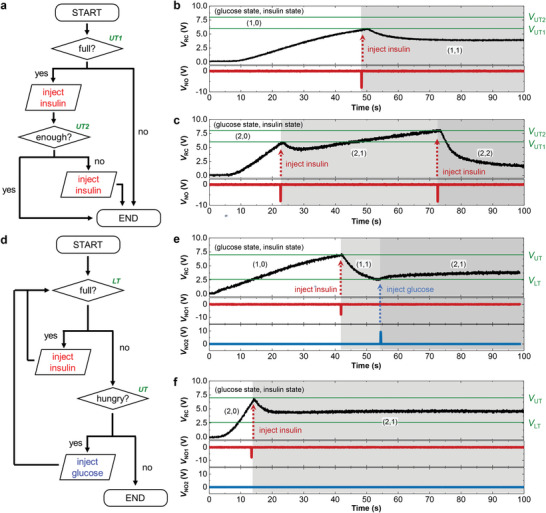
a) Logical algorithm of glucose‐level regulation system using double‐negative spike generation ANC. b,c) Real‐time *V*_RC_ and *V*_NO_ changes in the double‐negative spike generation ANC under an initial glucose state of b) 1 and c) 2. d) Logical algorithm of glucose‐level regulation system using complementary spike generation ANC. e,f) Real‐time *V*_RC_ and *V*_NO_ changes in the complementary spike generation ANC under an initial glucose state of e) 1 and f) 2.

Finally, glucose‐level‐regulating negative feedback loops were constructed by incorporating the functional sensors; SIC, AS, and ANC. Two types of glucose‐level‐regulating feedback loops, characterized by the abovementioned ANC, were demonstrated: a double insulin injection feedback loop, and an insulin–glucose injection feedback loop. **Figure** **4**a shows the logical algorithm of the double insulin injection feedback loop. Unless *V*_RC_ exceeds *V*_UT1_, no feedback regarding the glucose level is required. When *V*_RC_ reaches the first upper threshold (*V*_UT1_), which corresponds to an abnormal increase in the glucose level, the ANC outputs a negative pulse to promote insulin injection. After the first injection, *V*_RC_ can either remain in the range below *V*_UT1_ or further increase to reach the second threshold (*V*_UT2_). The former case indicates that the amount of injected insulin is sufficient to stabilize the glucose level. In the latter case, additional insulin is injected to induce a stronger suppression of glucose level increase, leading to glucose level stabilization. Figure 4b exemplifies the former case in which *V*_RC_ with an initial glucose state of 1 change according to the generated *V*_NO_. When the increasing *V*_RC_ exceeded *V*_UT1_, one dose of insulin was injected, and no additional injection is performed. The latter case with an initial glucose state of 2 is shown in Figure 4c. *V*_NO_ generated by the two thresholds effectively suppressed the rapid increase in *V*_RC_. The logical algorithm of the insulin–glucose injection feedback loop is shown in Figure 4d. When *V*_RC_ approaches *V*_UT_ or *V*_LT_ and crosses each threshold, the injection of insulin and glucose, respectively, is implemented to suppress the corresponding change. The regulation of *V*_RC_ by *V*_UT_ and *V*_LT_ is demonstrated in Figure 4e, successfully confining the level of *V*_RC_ to within the intended voltage range.

## 3 Conclusion

In conclusion, we designed a complementary control‐based self‐regulating artificial nervous system by adopting a barrier‐tunable SBT. To demonstrate the feasibility of the system, a blood glucose level regulation system was constructed by incorporating glucose/insulin sensors, SIC, AS, and ANC. The complementary signals received by each sensor modulate the corresponding Schottky barrier to be multiplexed into the positive and negative amplitudes of a single presynaptic signal. Next, the signal was transmitted to the AS to generate a PSC, which indicates the blood glucose level that reflects the demultiplexed contributions from each complementary signal. Finally, the ANC, which could be designed to have multiple modes, regulated the blood glucose level by deciding whether glucose and/or insulin injection must be implemented according to the PSC level. The proposed system capable of complementary control represents a pathway to enhance the sophistication and efficiency of artificial nervous systems.

## 4 Experimental Section

### Device Fabrication

To fabricate the single SBT and SIC, a 30‐nm‐thick ITO layer was deposited and patterned as a gate electrode onto a glass substrate conducting radio frequency magnetron sputtering and conventional PR photolithography process (AZ 5214E). After PR patterning, the ITO was chemically etched (35–37 vol% hydrochloric) and sintered in the furnace in ambient conditions (600 °C, 30 min). A 100‐nm‐thick SiO_2_ layer was deposited onto the ITO patterned glass substrate via the conventional sol–gel method to form the gate dielectric layer.^[^ ^43^ ^]^ ITO and IGZO were then deposited and patterned using the same processes as those of the gate electrode to form the metal contact and n‐type semiconductor layer, respectively. The IGZO layer was annealed at 300 °C for 2 h in the furnace and patterned through chemical etching with 3 vol% LCE‐12 solution (Cyantek Co). Next, monolayer graphene, grown by conventional chemical vapor deposition, was transferred using the polymeric supporting layer of poly(methyl methacrylate) and patterned via photolithography and subsequent reactive ion etching (RIE). The glucose/insulin sensor was fabricated using ITO source/drain electrodes and an IGZO semiconducting channel. The patterning PR blocking layer was formed to avoid direct contact of the chemicals with the ITO electrodes. To fabricate the AS, a P3HT solution with a concentration of 9 mg mL^−1^ in chloroform was prepared with stirring at 50 °C for 6 h. The prepared P3HT solution was then spin‐coated onto the Au‐prepatterned glass substrate (1500 rpm and 1 min) and dried overnight under an Ar condition. The deposited P3HT layer was patterned via the same process with graphene. Subsequently, an ion–gel precursor solution composed of poly(ethylene glycol) diacrylate (PEGDA), 2‐hydroxy‐2‐methylpropiophenone (HOMPP), and 1‐butyl‐3‐methylimidazolium hexafluorophosphate ([BMIM][PF_6_]) ionic liquid with a weight ratio of 2:1:22 was drop‐casted onto the Au/P3HT patterned substrate. UV irradiation was conducted to pattern the ion–gel layer through photo‐initiated crosslinking.

### ANC and Injection System Implementation

The ANCs were composed with of CMOS‐based commercial components: an operational amplifier (UA741CP, Texas Instruments), PNP and NPN BJTs (KSB1151YSTU, ON Semiconductor and TTC004BQ, Toshiba, respectively), resistors, and capacitors. The injection system of glucose and insulin solutions was implemented using a linear actuator (HJA301, Richmat).

### Characterization

The quality of graphene was analyzed through Raman spectroscopy (Alpha300M, Witec). The electrical properties of the single SBT, SIC, glucose/insulin sensor, and AS were evaluated using a Keithley 4200A‐SCS and vacuum probe station system. The real‐time output signals of ANC components were measured using a digital phosphor oscilloscope (DPO3052, Tektronix).

## Conflict of Interest

The authors declare no conflict of interest.

## Supporting information

Supporting Information

Supplemental Movie 1

## Data Availability

The data that support the findings of this study are available from the corresponding author upon reasonable request.
