# Supplementary material for: Multiplexed Complementary Signal Transmission for a Self‐Regulating Artificial Nervous System
Source: Adv Sci (Weinh). 2022 Nov 27;10(3):2205155. doi: 10.1002/advs.202205155 (PMC9875628; doi:10.1002/advs.202205155)
Supplement: Supplementary file 1 — Supporting Information [file ADVS-10-2205155-s002.pdf]

Supporting Information for

Multiplexed Complementary Signal Transmission for a Self-Regulating Artificial Nervous System

Young Jin Choi,[1,†] Dong Gue Roe,[2,†] Yoon Young Choi,[1] Seongchan Kim,[3] Sae Byeok Jo,[4] Hwa Sung Lee,[5] Do Hwan Kim,[6] Jeong Ho Cho[1,*]

[1]Department of Chemical and Biomolecular Engineering, Yonsei University, Seoul 120-749, Republic of Korea.
[2]School of Electrical and Electronic Engineering, Yonsei University, Seoul 03722, Republic of Korea.
[3]SKKU Advanced Institute of Nanotechnology (SAINT), Sungkyunkwan University, Suwon 16419, Korea.
[4]SKKU Institute of Energy Science and Technology (SIEST), School of Chemical Engineering, Sungkyunkwan University (SKKU), Suwon 16419, Republic of Korea.
[5]Department of Materials Science and Chemical Engineering, Hanyang University, Ansan 15588, Republic of Korea.
[6]Department of Chemical Engineering, Hanyang University, Seoul 04763, Republic of Korea.

*Corresponding author: J. H. Cho (jhcho94@yonsei.ac.kr)

[†]Y. J. Choi and D. G. Roe contributed equally this work.

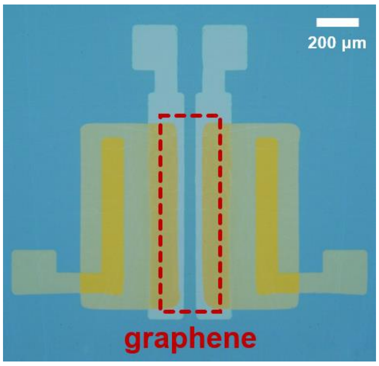

Figure S1. Optical microscopy image of the SIC.

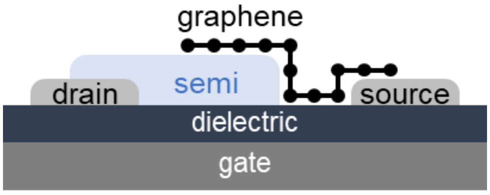

Figure S2. Schematic structure of graphene/n-type semiconductor heterojunction based SBT.

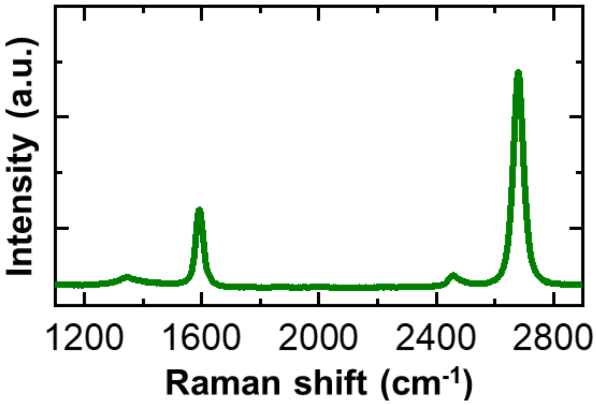

Figure S3. Raman spectrum of monolayer graphene, grown using the CVD method.

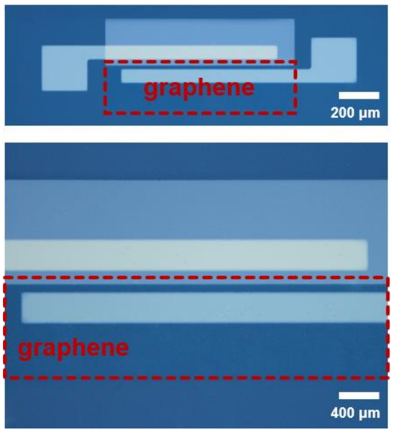

Figure S4. Optical microscopy image of graphene/IGZO based single SBT.

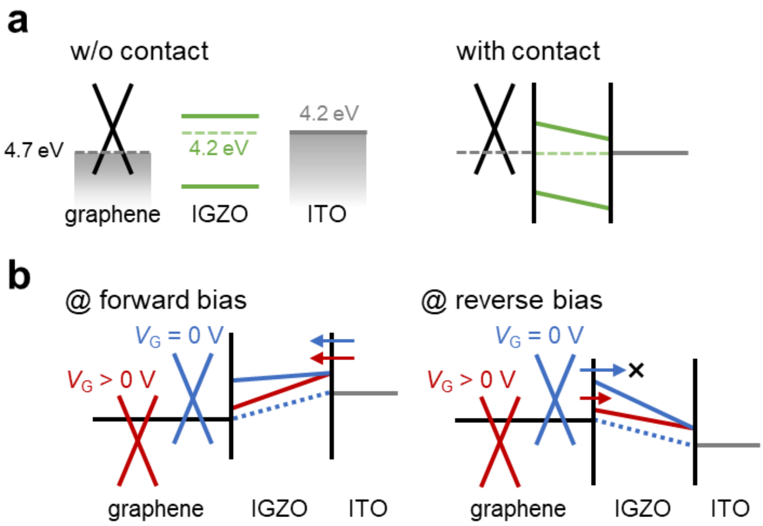

Figure S5. (a) Schematic band diagram of graphene, IGZO and ITO without and with contact. (b) Schematic band diagram of single SBT with V_G at forward and reverse bias conditions.

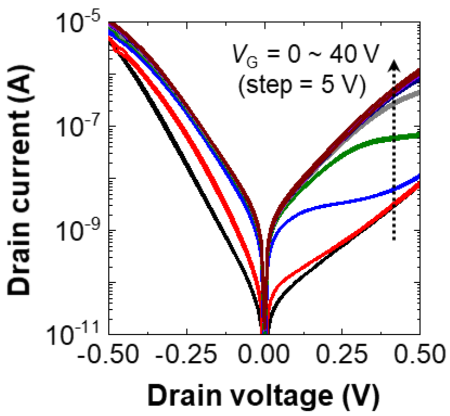

Figure S6. Output characteristic of single SBT under various V_G conditions.

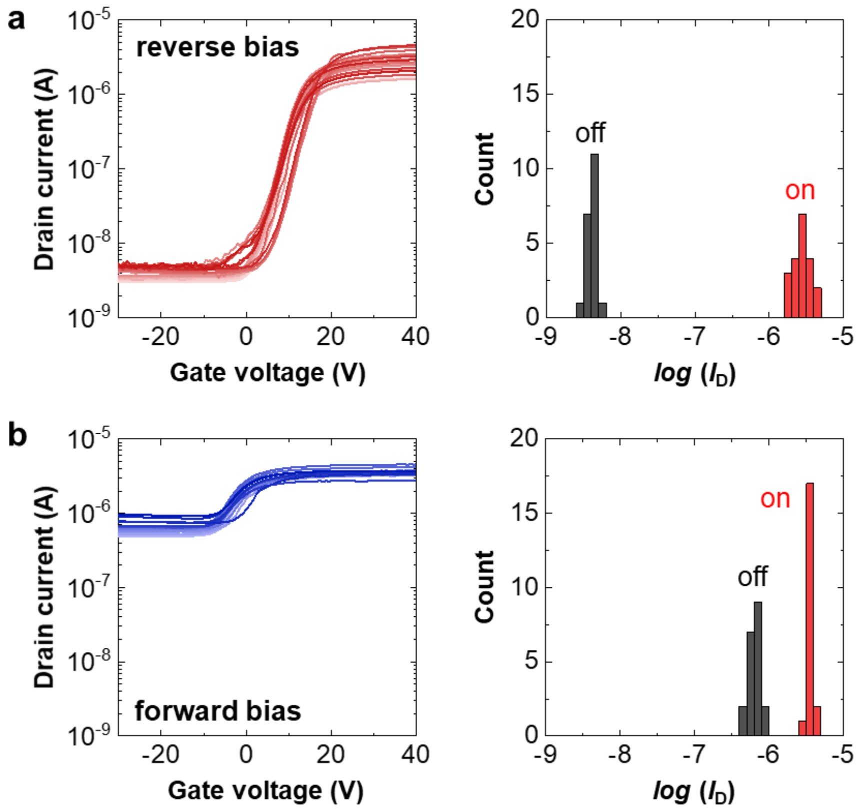

Figure S7. Transfer characteristics and histogram of 20 SBTs, under (a) reverse bias (V_D = 0.3 V) and (b) forward bias (V_D = −0.2 V).

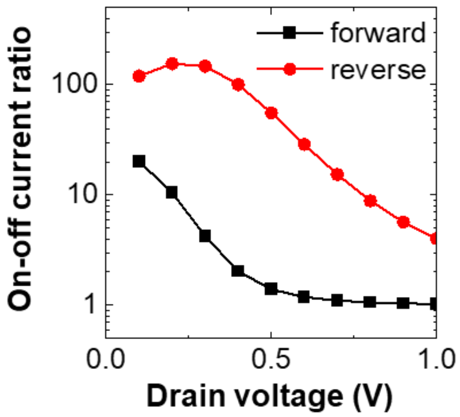

Figure S8. On-off current ratio versus V_D plots of forward and reverse bias conditions.

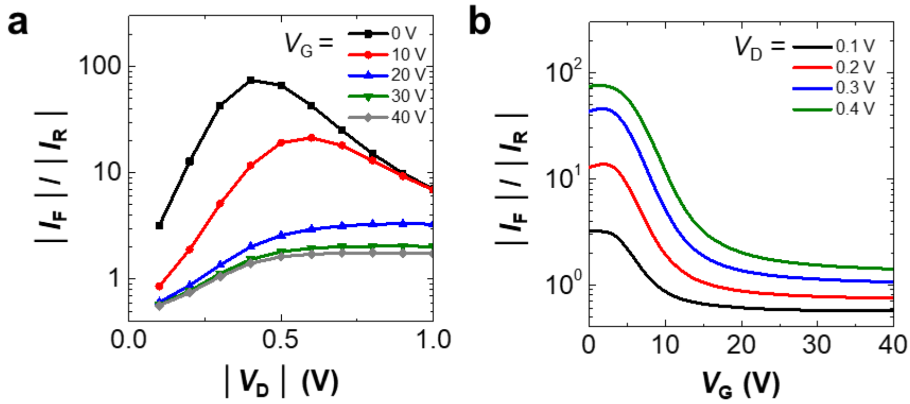

Figure S9. Change in the (a) | V_D | and (b) V_G dependent rectification ratio under different various bias conditions.

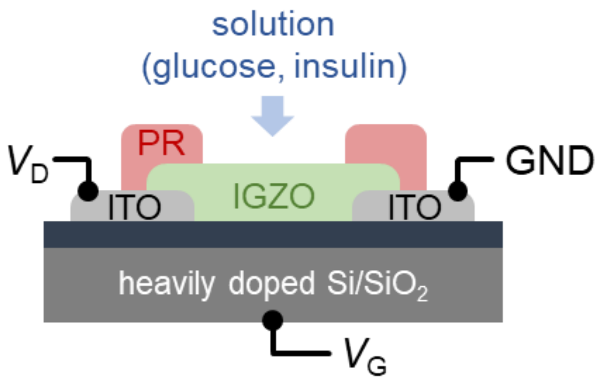

Figure S10. Schematic structure of IGZO based glucose/insulin transistor-type sensor.

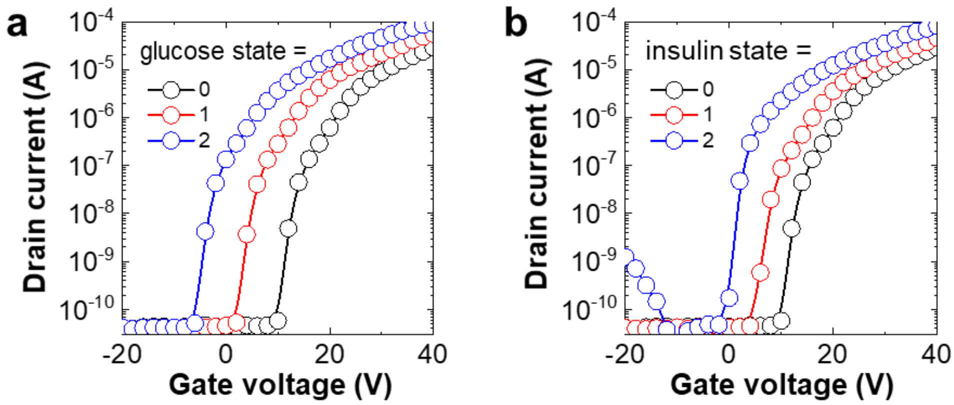

Figure S11. Transfer characteristics of IGZO based sensor under various (a) glucose and (b) insulin states.

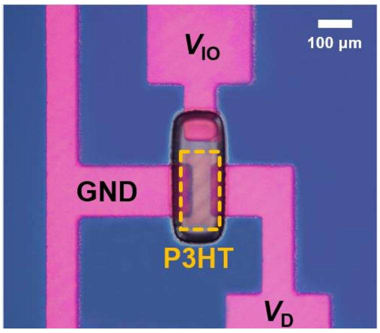

Figure S12. Optical microscopy image of P3HT/ion-gel based AS.

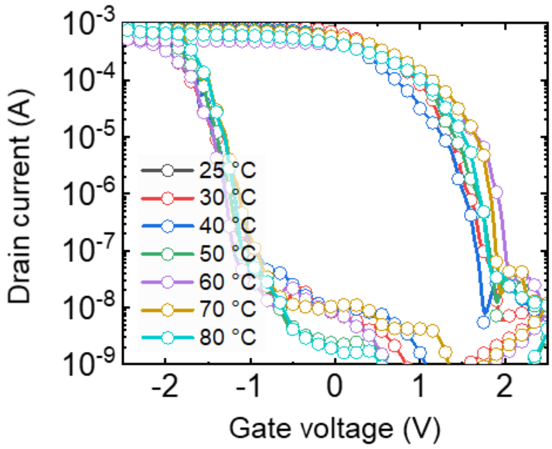

Figure S13. Transfer characteristics of AS with varying temperatures.

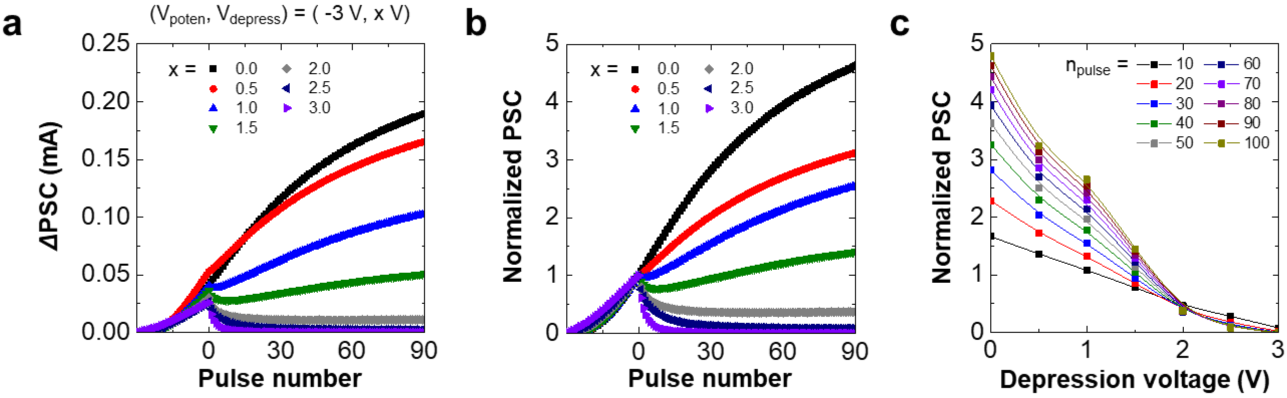

Figure S14. (a) ΔPSC and (b) Normalized PSC versus pulse number plots under an integrated pulse input with various depression voltage values. (c) Normalized PSC versus depression voltage curves for various pulse numbers under integrated pulse application.

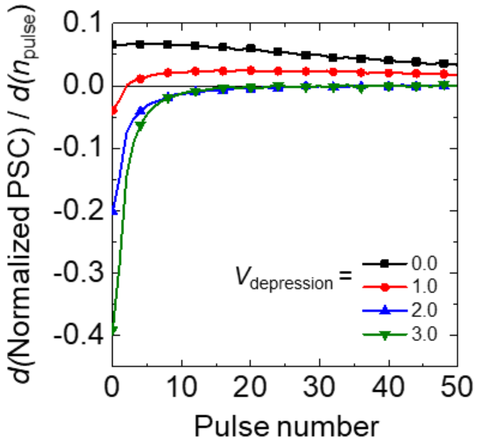

Figure S15. Derivative of normalized PSC under various depression voltages.

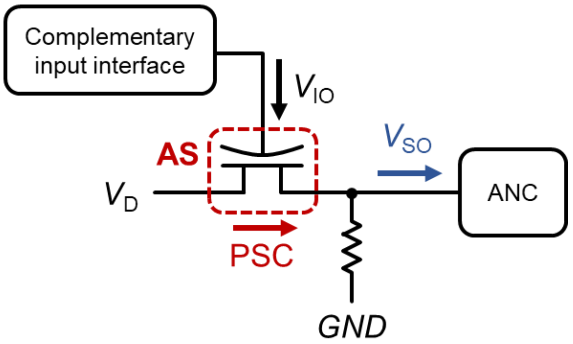

Figure S16. Schematic circuit diagram of a Complementary input interface, AS, and ANC, showing the signal conversion and transportations.

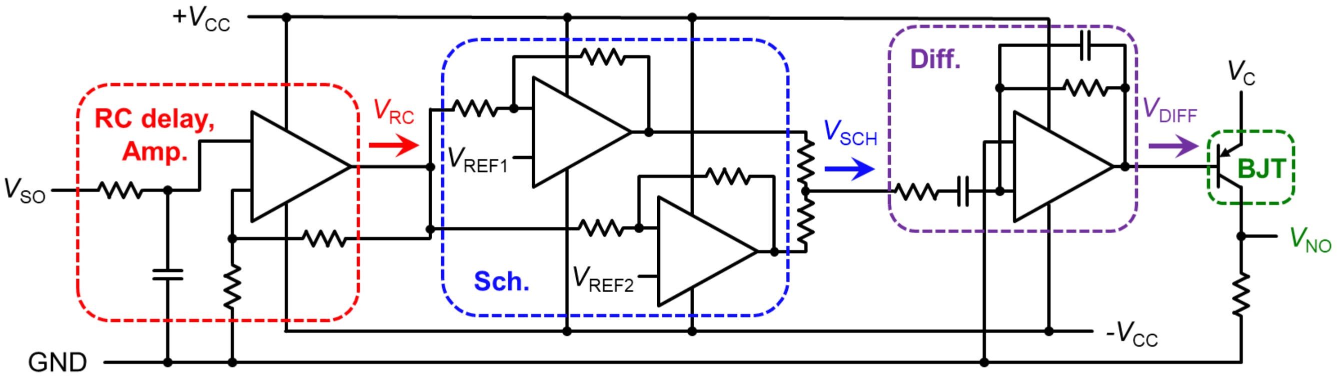

Figure S17. Circuit diagram of double-negative spike generation ANC.

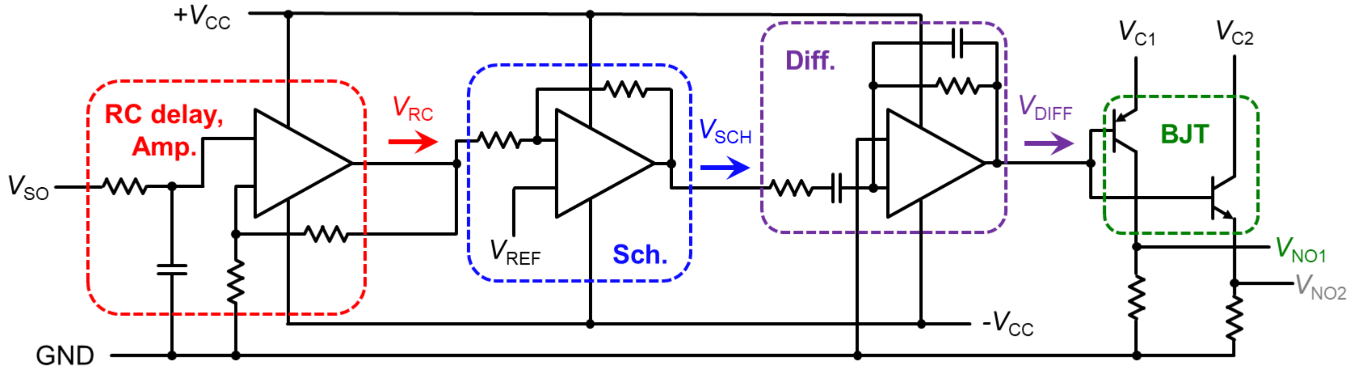

Figure S18. Circuit diagram of complementary spike generation ANC.

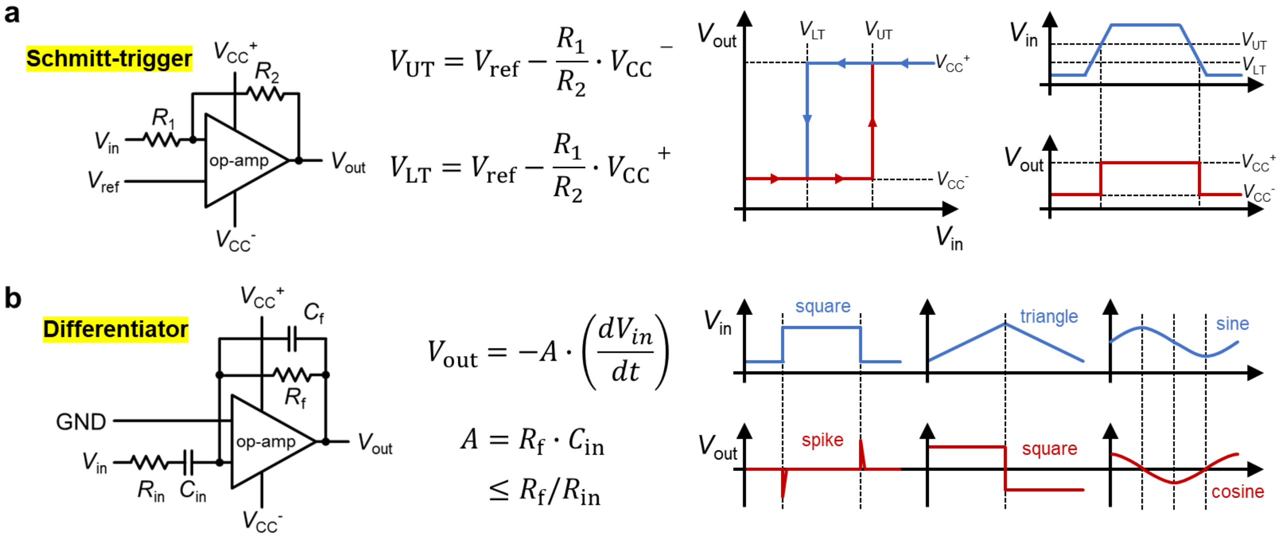

Figure S19. Circuit diagram, equations, and schematic input-output voltages of (a) Schmitt-trigger and (b) differentiator.

Movie S1. Demonstration of the real-time glucose-level regulation system.
